# Liquid Chromatography with a Fluorimetric Detection Method for Analysis of Paralytic Shellfish Toxins and Tetrodotoxin Based on a Porous Graphitic Carbon Column

**DOI:** 10.3390/toxins8070196

**Published:** 2016-06-28

**Authors:** Veronica Rey, Ana M. Botana, Mercedes Alvarez, Alvaro Antelo, Luis M. Botana

**Affiliations:** 1 Department Analytical Chemistry, Faculty of Sciences, University of Santiago de Compostela, Lugo 27002, Spain; veronica.rey@rai.usc.es; 2 CIFGA S.A., Plaza Santo Domingo 20-5ª, Lugo 27001, Spain; mercedes@cifga.es (M.A.); alvaro@cifga.es (A.A.); 3 Department Pharmacology, Veterinary Faculty, University of Santiago de Compostela, Lugo 27002, Spain

**Keywords:** paralytic shellfish toxins, tetrodotoxin, porous graphitic carbon, post-column oxidation liquid chromatography, shellfish matrices

## Abstract

Paralytic shellfish toxins (PST) traditionally have been analyzed by liquid chromatography with either pre- or post-column derivatization and always with a silica-based stationary phase. This technique resulted in different methods that need more than one run to analyze the toxins. Furthermore, tetrodotoxin (TTX) was recently found in bivalves of northward locations in Europe due to climate change, so it is important to analyze it along with PST because their signs of toxicity are similar in the bioassay. The methods described here detail a new approach to eliminate different runs, by using a new porous graphitic carbon stationary phase. Firstly we describe the separation of 13 PST that belong to different groups, taking into account the side-chains of substituents, in one single run of less than 30 min with good reproducibility. The method was assayed in four shellfish matrices: mussel (*Mytillus galloprovincialis*), clam (*Pecten maximus*), scallop (*Ruditapes decussatus*) and oyster (*Ostrea edulis*). The results for all of the parameters studied are provided, and the detection limits for the majority of toxins were improved with regard to previous liquid chromatography methods: the lowest values were those for decarbamoyl-gonyautoxin 2 (dcGTX2) and gonyautoxin 2 (GTX2) in mussel (0.0001 mg saxitoxin (STX)·diHCl kg^−1^ for each toxin), decarbamoyl-saxitoxin (dcSTX) in clam (0.0003 mg STX·diHCl kg^−1^), *N*-sulfocarbamoyl-gonyautoxins 2 and 3 (C1 and C2) in scallop (0.0001 mg STX·diHCl kg^−1^ for each toxin) and dcSTX (0.0003 mg STX·diHCl kg^−1^ ) in oyster; gonyautoxin 2 (GTX2) showed the highest limit of detection in oyster (0.0366 mg STX·diHCl kg^−1^). Secondly, we propose a modification of the method for the simultaneous analysis of PST and TTX, with some minor changes in the solvent gradient, although the detection limit for TTX does not allow its use nowadays for regulatory purposes.

## 1. Introduction

Marine biotoxins are produced by more than 200 marine algal species. They are complex secondary metabolite molecules with high toxicity and an unknown physiological role. The intake of these compounds can lead to serious toxic effects in humans, including death [1,2].

Paralytic shellfish toxins (PST) cause persistent problems in humans due to their accumulation in filter feeding shellfish [3], but they can also move up through the food chain, affecting zooplankton, fish, birds and marine mammals [4]. PST intoxications are a result of exposure to saxitoxins (STXs), gonyautoxins (GTXs) and *N*-sulfocarbamoyl-gonyautoxins (Cs). More than 57 different analogues of saxitoxin (STX) have been described to date [5,6].

Analytical methods can be used to detect and quantify PST. High performance liquid chromatography (HPLC) was one of the first analytical methods developed for STX detection, and it is routinely used. The basis of the HPLC method for PST analysis was established in the late 1970s, using post-column derivatization with a silica-based stationary phase [7]. Since then, many methods for toxin separation have been developed using both pre- and post-column oxidation with different types of columns. A variety of modifications have led to improved separation and detection of the different congeners, including sample extraction, type of column, eluent composition and oxidation processes [8]. The most common chemical method uses a combination of HPLC with either pre- or post-column oxidation followed by fluorescence detection (FLD) [9,10]. The Lawrence method has been validated by the Association of Official Analytical Chemists (AOAC) through a collaborative trial [11] and was adopted as the First Action HPLC-FLD Official Method [12]. It involves separation of the PST after their derivatization and further fluorescence detection. The original method was refined and extended to include other toxins in mussel and other matrices [13,14,15]. The major challenge of the pre-column oxidation method is that the chromatographic separation is performed on the oxidized reaction products. Many of the toxins have reaction products with more than one peak, namely, decarbamoyl-GTX2,3 (dcGTX2,3), neosaxitoxin (NEO), GTX6, GTX1,4, C3,4, dcSTX and dcNEO, and some of them coelute after the periodate oxidation. This happens for the two peaks of both dcSTX and dcNEO, the second peak of GTX1,4 (with three peaks) and of dcGTX2,3, the third one of GTX1,4 and the single peak of GTX2,3. Therefore, a misidentification and wrong quantification of several PST could take place [16]. Moreover, this method is unable to separate pairs of isomers; hence, the total toxicity of the samples is overestimated by using the highest toxicity equivalency factor (TEF) [17,18,19] for those isomers that yield the same coeluting oxidation products.

The post-column technique published by Oshima [20] was modified by Thomas et al. [10] and then further refined by Rourke et al. [21]. These modifications included different clean-up procedures, eluent composition, HPLC columns and oxidation conditions. Nevertheless, it is necessary to perform two analyses with two different columns to identify and quantify the whole variety of PSTs.

The post-column oxidation (PCOX) method has undergone a single laboratory validation [22] and a collaborative validation [23], and it was adopted as an official AOAC method [24]. The advantage of this method is that it enables quantification and isolation of each epimer individually and shows a good correlation with the mouse bioassay; on the contrary it requires additional time to change columns, and the column lifetime is shortened due to the use of ion pairing reagents in mobile phases; it also shows interferences of naturally fluorescent compounds present in matrices, as Biré et al. [25] remarked. Furthermore, Boundy et al. [26] have recently developed a hydrophilic interaction ultra-performance liquid chromatography tandem mass spectrometry (HILIC UPLC-MS/MS) method for PST that includes an optimized desalting clean-up procedure using carbon solid phase extraction cartridges to reduce matrix interferences.

In addition to all of the above, tetrodotoxin (TTX), has been reported in Greek and English bivalves [27,28], and it has the same toxicity symptoms and mechanism of action as PST [29]. TTX is a potent neurotoxin with a low molecular weight, is water soluble and heat stable; therefore cooking does not reduce its toxicity, but it may increase its toxic effect [30]. It frequently causes intoxications due to the ingestion of puffer fish, very common in Japan [31]. Although TTX exists mainly in tropical waters around the world, recent studies have demonstrated that it has appeared on the European coasts and up to England, due to climate change [28]. This is possibly due to “Lessepsian migration”: the opening of the Suez Canal caused the migration of many Red Sea species to colonize the Mediterranean Sea [32].

After a serious intoxication with TTX by ingestion of gastropods [33], it was clear that TTX was a food safety concern in Europe. Furthermore, the co-occurrence of TTX with PST has been reported in many species of puffer fish, crabs and gastropods [34]. Currently, there is no official method for detecting these toxins; however, the mouse bioassay has been used in many cases to determine their toxicity [35]. Since they block sodium channels in a similar fashion as STX [36], their presence in seafood would be misdiagnosed by the mouse bioassay [29]. To obtain specific information from a sample, such as the toxin profile or the amount of a single analogue, chemical methods have been developed based on HPLC-FLD [37]. Although these methods ensure low detection limits, liquid-chromatography-mass spectrometry (LC-MS) and especially LC-MS/MS [29] are generally regarded as the best choice for the determination of TTX and related compounds.

Regulatory limits have not been established for this toxin and its analogues [38]. The European Food Safety Authority (EFSA) has not published any document regarding risks from TTX, but some authors affirm that the minimum toxic dose in humans is 2 mg [31,39].

This work proposes a new method to determine simultaneously all PST with a porous graphitic carbon column using HPLC-FLD. The method proposes also a modification for the separation of PST alongside TTX. We proved that it was possible to analyze all of these toxins together and in a short time, compared to other chromatographic methods.

## 2. Results and Discussion

### 2.1. Chromatographic Conditions

Although the initial chromatographic conditions for PCOX separation of PSTs were those reported by Van de Riet et al. [22], several modifications were accomplished: the column and mobile phase were different. While in PCOX the separation is based on the use of heptane sulfonate and tetrabutyl ammonium phosphate, in this case, the mobile phase was based on the use of trifluoroacetic acid (TFA). The post-column conditions were kept with slight modifications: the oxidant was the same, but the flow rate was different (0.4 mL·min^−1^ in PCOX and 0.5 mL·min^−1^ in this work); the concentration of nitric acid was also changed, from 0.75 M at a flow rate of 0.4 mL·min^−1^ to 0.1 M at a flow rate of 0.3 mL·min^−1^. These modifications in acid concentration and flow rate and in the oxidant flow rate were performed to achieve a final outflow pH in the range between five and seven [17]. Several parameters were tested to obtain the best sensitivity and resolution. The initial conditions were: 0.025% TFA in Solvents A and B, column temperature 15 °C, reaction temperature 80 °C and outflow pH = 6.

#### 2.1.1. Development of TFA as an Ion Pairing Agent

**Figure 1:**
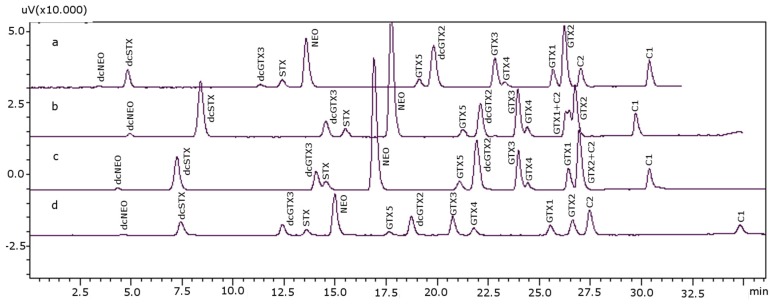
Chromatographic separation of paralytic shellfish toxins (PST) standards using different percentages of trifluoroacetic acid (TFA) in the mobile phase (the analysis time is not optimized for all of the parameters of the method): (**a**) 0.025% TFA in Solvents A and B; (**b**) 0.05% TFA in Solvents A and B; (**c**) 0.075% TFA in Solvents A and B; (**d**) 0.075% TFA in Solvent A and 0.025% in Solvent B. Concentrations used for the standards (as mg saxitoxin (STX)·diHCl eq·kg^−1^): 0.1387 for decarbamoyl-neosaxitoxin (dcNEO), 0.1337 for dcSTX, 0.0018 for dc gonyautoxin 3 (dcGTX3), 0.4182 for STX, 0.6761 for NEO, 0.0020 for GTX5, 0.0045 for dcGTX2, 0.0523 for GTX3, 0.0016 for GTX4, 0.0080 for GTX1, 0.0419 for GTX2, 0.0002 for C1 and 0.0012 for C2.

Once the appropriate gradient for separating all toxins was achieved, the percentage of TFA was then tested. TFA can act as a competitive electronic modifier in the mobile phase to change the polar retention. It forms an ion pair with the analyte, increasing retention times for most of the toxins when its concentration was increased: dcNEO was delayed 1–1.5 min; dcSTX, dcGTX2, dcGTX3, STX, NEO and GTX5 were delayed 3–3.5 min; GTX2, GTX3, GTX1 and GTX4 were delayed 1 min; C1 and C2 were advanced 0.5–1 min (Figure 1). On the other hand, the resolution and peak shape were also improved through electronic interactions with the graphite surface. The porous graphite column stability allows high concentrations of aggressive buffers, such as 1% TFA, to be used with no detrimental effect on the column lifetime, but when the concentration of TFA is increased beyond 0.1%, there is a significant effect on the peak shape and resolution of the basic analytes, according to manufacturer indications [40]. Therefore, different percentages of TFA in the mobile phase for both Solvents A and B were assayed: specifically, 0.025%, 0.050% and 0.075%, while column temperature, reaction temperature and outflow pH remained at their initial conditions (15 °C, 80 °C and six, respectively).

At this stage, the interpretation of the quality of separation was based on the resolution between adjoining peaks and their symmetry. A concentration of 0.025% TFA was ideal for the separation of the last eluting toxins, although this low percentage of TFA decreased the peak resolution of earlier eluted toxins (dcNEO, dcSTX, NEO, STX and dcGTX3), as well as the symmetry and number of theoretical plates; therefore, higher amounts of TFA (0.075%) were necessary to improve these parameters. According to this, different combinations of concentrations for Solvents A and B were tried, and the best performing mixture was 0.075% TFA in Solvent A and 0.025% TFA in Solvent B; these results are shown in Figure 1 (in this figure, the optimized analysis time is not shown). The improvement in the separation obtained with TFA for the di-cationic PST (dcNEO, dcSTX, STX and NEO) seemed to be related to the role that TFA plays as an ion-pair reagent and not only to the acidification of the medium. Therefore, the TFA percentage was an important issue to take into account.

#### 2.1.2. Detector Outflow pH

Once the optimal mobile phase composition was accomplished, the effect of pH on the oxidation of PST was studied to establish the conditions that produced the maximum fluorescence in the oxidized products (column and reaction temperature remained the same). Vale et al. [17] suggested that the outflow pH should be in the range between five and seven; in order to get a value in this range, several combinations of oxidant and acid flow rates were tested, and the working outflow pHs were 5, 6 and 7. The best resolution was obtained at pH = 5. To carry out these tests, both column temperature and reaction temperature were not modified, and %TFA was 0.075% in Solvent A and 0.025% in B, as was described in the previous section. These conditions allowed a good separation in an acceptable run time.

#### 2.1.3. Reaction Temperature

**Figure 2:**
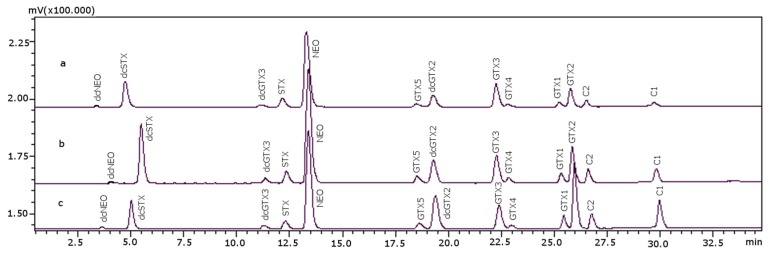
Chromatograms of PST standards obtained at different reaction temperatures (the analysis time is not optimized for all of the parameters of the method): (**a**) 65 °C; (**b**) 75 °C; (**c**) 85 °C. Concentrations used for the standards (as mg STX·diHCl eq·kg^−1^): 0.1387 for dcNEO, 0.1337 for dcSTX, 0.0018 for dcGTX3, 0.4182 for STX, 0.6761 for NEO, 0.0020 for GTX5, 0.0045 for dcGTX2, 0.0523 for GTX3, 0.0016 for GTX4, 0.0080 for GTX1, 0.0419 for GTX2, 0.0002 for C1 and 0.0012 for C2.

The reaction temperature was optimized while column temperature remained at 15 °C, its initial value; this working temperature depends on the type of heat source, water bath or reactor [20]. As the equipment setup was attached to an online water bath, the following temperatures were assayed: 65 °C, 70 °C, 75 °C, 80 °C and 85 °C. The temperature that produced the peaks with the highest signal:noise ratio was 75 °C. Figure 2 shows the chromatograms at some of those temperatures: dcNEO, dcSTX, dcGTX3, GTX5 and GTX4 peaks are bigger at 75 °C than at other temperatures; this is quite important in the case of dcNEO due to its low signal and also for dcGTX3, GTX5 and GTX4. Comparison of peaks at 65 °C and the optimal temperature 75 °C gave the following: dcNEO, dcGTX3 and GTX4 were 40% higher at 75 °C than at 65 °C; dcSTX and GTX5 were 50% higher at the optimal temperature than at 65 °C. The rest of the peaks increased their height between 15% and 30%.

#### 2.1.4. Column Temperature

**Figure 3:**
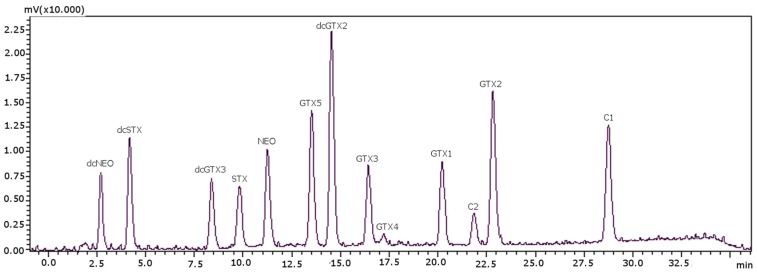
Chromatographic separation of PST standards with the post-column oxidation method, using a porous graphitic carbon column. Concentrations used for the standards (as mg STX·diHCl eq·kg^−1^): 0.6780 for dcNEO, 0.4354 for dcSTX, 0.0793 for dcGTX3, 1.0634 for STX, 1.6910 for NEO, 0.0956 for GTX5, 0.1062 for dcGTX2, 0.2107 for GTX3, 0.0160 for GTX4, 0.0858 for GTX1, 0.1656 for GTX2, 0.0021 for C1 and 0.0248 for C2.

Column temperature was the last parameter to be optimized. In this method, C1 and C2 toxins and GTXs and STXs groups were analyzed in one single run, whereas the PCOX method [22] required two columns for the same purpose, one for Cs with temperature below room conditions to prevent on-column epimerization of C1 and C2 and another one for the rest of the toxins. Therefore, the optimal column temperature was investigated for all of the toxins in a range from 15 °C (minimal oven temperature) and up to 35 °C. At 20 °C, the peak signals showed the highest values: for temperatures above 20 °C, the peaks size decreased, and the retention times decreased, resulting in the overlapping of both dcSTX and dcNEO, between them and with the solvent front. Therefore, 20 °C was established as the best temperature, and the optimized separation is shown in Figure 3.

### 2.2. Extraction and Sample Clean-Up

**Figure 4:**
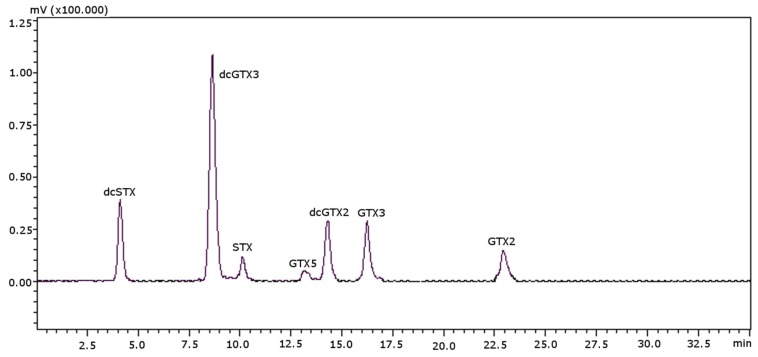
Chromatographic separation of PST in a real scallop sample using a porous graphitic carbon column. Concentrations used for the standards (as mg STX·diHCl eq·kg^−1^): 0.2857 for dcSTX, 0.3217 for dcGTX3, 1.6801 for STX, 0.0406 for GTX5, 0.0476 for dcGTX2, 0.0401 for GTX3, 0.3280 for GTX2.

The aim of this work was to establish the validation parameters of the method for the column; therefore, the samples used must not be contaminated with either PST or TTX. A contaminated scallop sample is shown in Figure 4, as an example of the separation of PST with this column for the optimized conditions.

PSTs were extracted using the AOAC mouse bioassay (MBA) method [41]. Many authors [42,43,44] have suggested that the MBA extraction with HCl 0.1 M is useful in monitoring programs, because it detects the composite toxicity in samples, while the acetic acid extraction (Lawrence method) is more suitable for studying the toxin profile in samples. The main disadvantage of HCl extraction [42,43] is that *N*-sulfocarbamoyl toxins are easily converted under mild acid treatment and heat into their corresponding carbamate toxins. Acetic acid extracts have a pH between 4 and 4.5, while the pH of the hydrochloric extracts is three; the recovery of these extracts at pH = 3 during SPE-C18 clean-up increases the toxicity in the HCl extraction [17,21,45]. In order to maintain the essence of the PCOX extraction, the hydrochloric extraction procedure was chosen in this work.

### 2.3. Validation Parameters

Table 1 shows the linearity results for each individual paralytic shellfish toxin. All of the correlation coefficients were higher than 0.99 (except one with 0.985). Peak areas were linear between the following lowest and highest concentration values: 0.117–16.003 mg STX·diHCl eq·kg^−1^, respectively, for all toxins. The range of linearity obtained was not as broad as in the PCOX method; nevertheless, the regulatory limit of 0.8 mg STX·diHCl eq·kg^−1^ is well within this range.

Table 2 shows limit of detection (LOD) values for PSTs in mussel, clam, scallop and oyster. These values ranged from 0.0001 to 0.0366 mg STX·diHCl eq·kg^−1^.The lowest values were those for dcGTX2 and GTX2 in mussel and C1 and C2 in scallop; GTX2 showed the highest LOD in oyster, being 0.0366 mg STX·diHCl eq·kg^−1^. These results were better than those obtained for the PCOX method in our laboratory [46], where LODs ranged from 0.0001 mg STX·diHCl eq·kg^−1^ for C1 in mussel matrix to 0.057 mg STX·diHCl eq·kg^−1^ for GTX1 in oyster matrix.

Limits of quantification (LOQs) are also summarized in Table 2, ranging from 0.0001 to 0.0750 mg STX·diHCl eq·kg^−1^. The values for dcNEO in oyster and in scallop were the highest, which might be due to the low intensity of the toxin fluorescence signal response.

For the majority of toxins, the values obtained were improved with regard to those reported by previously published LC-fluorescence methods [11,22]; therefore, they showed a good detection and quantitation capability for regulatory purposes.

The repeatability of the method for each toxin was calculated, and the results are summarized in Table 3. The RSD percentages (%RSD) for all toxins were within the acceptable range, as indicated by IUPAC (International Union of Pure and Applied Chemistry) [47]. The highest variations appeared in dcNEO for all tested matrices at low concentrations, which can be due to its low fluorescence signal. The rest of the toxins gave relatively consistent values, except for the oyster matrix, which showed the highest variations. Despite this, replicate injections of sample tissue extract solutions indicated good peak response repeatability over the range of concentrations studied; the %RSD ranged from 1.6%–9.4%.

Retention times were stable, and the %RSD varied from 0.1%–0.9% (Table 4). The order of elution for C2 and GTX2 toxins changed after column regeneration: initially, the elution order was GTX2 and C2 (as can be seen in Figure 1 and Figure 2), and after regeneration, the order of elution was inverted: C2 eluted before GTX2, and it remained the same after successive regenerations (Figure 3). Approximately 600 samples could be analyzed before the regeneration of the column, and a total of 6000 injections were possible, which is 10-times more than what is possible with PCOX columns. Although it could be seen as a lack of reproducibility and reliability of the column, Table 4 shows that the %RSD of retention times were very low. Therefore, it should not be considered as a lack of reproducibility, but since this may be perceived as a source of confusion, an easy solution would be to treat the column before its first use with a regenerative process, since it does not shorten its working life. Figure 3 shows the separation accomplished for PST after regeneration of the column. All of the validation parameters obtained in this work were made with the regenerated column. After this treatment, the elution order was stable. We also tested the validation parameters in a new column before regeneration, and all of the values matched those obtained after the regeneration.

Some guidance documents request the determination of the recovery for the lowest and highest concentrations [48,49] or even specify that the recovery should be above 50% [48]. According to other authors [50], the acceptable range of recovery depends on the concentration; in this study, the concentration varied between 0.02 mg·kg^−1^ and 0.1 mg·kg^−1^, and the acceptable mean recovery percentage range was 70%–120%. Table 5 summarizes the mean recovery percentages of PST in mussel, clam, scallop and oyster matrices. The recoveries of dcNEO and dcGTX3 in all matrices were circa 67% and 66%, respectively, which were slightly below the values for the rest of the toxins. Moreover, the matrix that gave the lowest results was oyster, whose values for six toxins were slightly below the acceptable range established between 70% and 120%. Very important, along with the values of recovery, is the repeatability, and the observed %RSD data values indicated that it was satisfactory.

**Figure 5:**
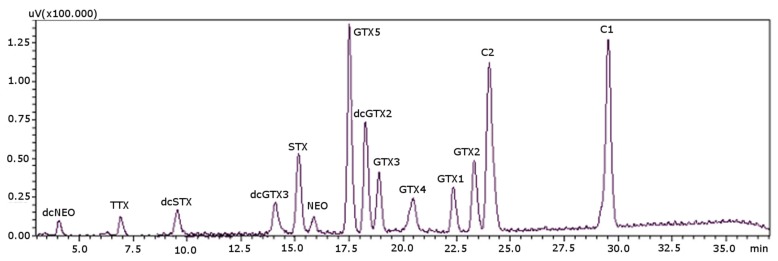
Chromatographic separation of PST and tetrodotoxin (TTX) standards with the post-column oxidation method, using a porous graphitic carbon column. Concentrations used for the PST standards (as mg STX∙diHCl eq·kg^−1^): 0.0978 for dcNEO, 0.0251 for dcSTX, 0.0040 for dcGTX3, 0.3945 for STX, 0.4265 for NEO, 0.0017 for GTX5, 0.0005 for dcGTX2, 0.0277 for GTX3, 0.0020 for GTX4, 0.0094 for GTX1, 0.0249 for GTX2, 0.0002 for C1 and 0.0010 for C2; concentration of TTX standard (μM): 68.6.

In addition to validation information about PST analysis with a porous graphite column and the improvement that implies its use to analyze PST, we also propose a modification of this method to include the analysis of TTX because of its appearance in marine environments together with those toxins. Although it could be adequate to propose only one method to analyze PST and TTX, we think that it is more convenient to have one method for PST and a modification of it to include TTX, because they belong to different groups of toxins. Figure 5 shows the separation of all toxins in one analysis, where the gradient elution had to be changed with regard to PST separation, as is described in the Materials and Methods (Section 4.4). In this way, two methods are developed that allow one to determine either PST or PST along with TTX.

For TTX, the recovery percentage, as well as LOD and LOQ were calculated for spiked mussel matrix. The recovery percentage (74.7% ± 1.5%) was determined analyzing five replicates of spiked mussel matrix: it was calculated at one level of concentration (16.747 mg·kg^−1^) that was approximately in the middle of the linearity range. The linearity range was between 10.306 and 21.900 mg·kg^−1^ (*r* = 0.9953). LOD and LOQ values (in µg·mL^−1^) were 3.7957 and 10.7304, respectively.

LOD and LOQ data were higher than those obtained by LC-MS/MS, where they are in the range of ng·mL^−1^ [29], and this was due to the low fluorescence signal of TTX. It is possible to improve the limit of detection for TTX with the use of a more sophisticated fluorescence detection system (i.e., with double excitation fluorimeters); although, as we report now, LOD and LOQ of TTX do not allow using this method for TTX regulatory purposes. Nonetheless, it shows that all toxins can be separated and detected in one single injection, improving considerably the current methods. The recovery percentage was within the acceptable range (70%–120%) [50], and although TTX is being found in new matrices, the matrix used in this work was mussel, because it was amenable to contain both PST and TTX, as already reported [27].

In previous works [51,52], TTX and PST were identified simultaneously by using the post-column oxidation method [20], but as happens with PST, they were not analyzed in one single injection. In this work, the only modification regarding the method proposed for PST was the variation in gradient program, because otherwise, dcNEO and TTX would coelute. Therefore, 14 toxins, belonging to two different groups, were separated and analyzed: PST and TTX. The run time of 37 min (which includes the 7-min equilibration time) is suitable for a chromatographic separation, and in this way, the possibility of misidentification for TTX in regard to PST was sorted out. As there is no commercial certified standard available for TTX analogues, it was not possible to analyze them.

## 3. Conclusions

This work describes a liquid-chromatography method with fluorescence detection to analyze PSTs and a variation to analyze also TTX and PST. The first one allowed the separation and quantitation of all PST available as commercial standards, in one single run and in an adequate time: 13 analogues were analyzed in 37 min. It was based on the use of a porous graphitized column with good properties related to separation performance and robustness. This method contributes to improve the analytical methodology for separation and quantitation of PST.

The second method was a modification of the first one, where the gradient elution was changed, allowing one to see in one run 13 commercial standards of PST alongside TTX. This improved the separation of those toxins, being the first time that they were analyzed all together in one single run. This method enables one to analyze them more easily due to the more frequent appearance of TTX in marine waters, where they were not seen before, because of global warming.

## 4. Materials and Methods

### 4.1. Chemicals

HPLC-grade methanol and acetonitrile, analytical reagent-grade sodium hydroxide, periodic acid, hydrochloric acid 37%, ortho-phosphoric acid 85%, nitric acid 65%, trifluoroacetic acid (TFA), dichloromethane, tetrahydrofuran and ammonium acetate were all from Panreac Química S.A., Barcelona, Spain. Trichloroacetic acid and triethylamine were purchased from Sigma Aldrich (Madrid, Spain). Only HPLC-grade water was used to prepare reagent solutions.

Certified standards provided by Cifga S.A. (Lugo, Spain) were: saxitoxin (STX), neosaxitoxin (NEO), decarbamoyl-saxitoxin (dcSTX), gonyautoxin 5 (GTX5), gonyautoxins 1 and 4 (GTX1,4), gonyautoxins 2 and 3 (GTX2,3), decarbamoyl-gonyautoxins 2 and 3 (dcGTX2,3), *N*-sulfocarbamoyl-gonyautoxins 2 and 3 (C1 and C2) and tetrodotoxin (TTX); decarbamoyl-neosaxitoxin (dcNEO) was obtained from NRC (Halifax, NS, Canada).

Commercial toxin-free shellfish samples of mussel (*Mytillus galloprovincialis*), clam (*Pecten maximus*), scallop (*Ruditapes decussatus*) and oyster (*Ostrea edulis*) were used.

### 4.2. Instrumentation

The chromatographic setup from Shimadzu (Izasa, Barcelona, Spain) included a binary LC-10ADVP pump system, autoinjector SIL-20AC with a refrigerated rack at 6 °C, column oven CTO-20AC, a fluorescence detector RF-10AXL and the system controller CBM-20A. The post-column reaction system was formed by a knitted reaction coil of 1 mL (5 m × 0.50 mm i.d., Supelco, Madrid, Spain) immersed in a water bath at 75 °C and two post-column pumps LC-20AD and LC-6A, respectively, from Shimadzu (Izasa, Barcelona, Spain). The separation and identification of toxins was achieved in a porous graphitic carbon Hypercarb^®^ column (i.d. 4.6 mm × 100 mm, 5-µm particle size, Part Number 35005-104630) from Thermo (Fisher Scientific, Madrid, Spain), inside the column oven at 20 °C.

### 4.3. Samples Extraction and Clean-Up

The protocol according to the PCOX method [22] was applied for the extraction of PST from shellfish material. A 5-g sample was homogenized with 5 mL of 0.1 M HCl using a vortex mixer. The pH of the mixture, which should be, according to the official protocol, between 2 and 4, was adjusted to 3 [41]. If necessary, the pH was adjusted while mixing by adding dropwise 5 M HCl to lower the pH or 0.1 M NaOH to raise the pH. The tube was placed in a boiling water bath for 5 min, removed and allowed to cool down to room temperature. The pH was again adjusted to 3 when necessary. The mixture was centrifuged at 3000× *g* for 10 min and the supernatant decanted.

Samples were deproteinated by adding 25 µL of 30% (*w*·*v*^−1^) trichloroacetic acid to 500 µL shellfish extract, mixed in a vortex and centrifuged at 16,000× *g* for 5 min. Then, 40 µL of 1.0 M NaOH were added, mixed and centrifuged at 16,000× *g* for 5 min [22]. The sample was then extracted with 500 µL of dichloromethane, mixed in a vortex and the aqueous and organic phases separated by gravity.

The aqueous phase was cleansed using a Hypersep Hypercarb^®^ (PGC) SPE cartridge (200 mg 3^−1^mL^−1^) (Thermo Fisher Scientific, Madrid, Spain), which was previously conditioned with 3 mL of 80% (*v*·*v*^−1^) dichloromethane:methanol, 3 mL of methanol and 3 mL of 0.1 M ammonium acetate. A 500-µL extract was then loaded and washed with 11 mL of 0.1 M ammonium acetate. The PST were eluted with 500 µL of citrate buffer in 10% (*v*·*v*^−1^) acetonitrile.

The same extraction and clean-up procedures were used when TTX was analyzed, and for this toxin, the seafood material was only mussel matrix.

### 4.4. Development of HPLC and PCOX Fluorescence Detection

The key parameters to be controlled were: the oxidant flowrate, acid flowrate, column temperature, reaction temperature, percentage of TFA and percentage of organic modifier. They were all tested to improve peak shape, resolution and sensitivity.

The final conditions recommended for routine operation were the following: all of the toxins were separated using a 100 mm × 4.6 mm i.d., 5-µm Hypercarb^®^ column (Thermo, Fisher Scientific, Madrid, Spain) with the column oven at 20 °C. Solvent A is 0.075% (*v*·*v*^−1^) TFA in water; Solvent B is 0.025% (*v*·*v*^−1^) TFA in 50% (*v*·*v*^−1^) acetonitrile:water. The flowrate was set at 0.8 mL·min^−1^, and the injection volume was 5 µL. The gradient conditions for PST were: start at 4% B, 4%–25% B in 30 min (linear slope), back to 4% B at 30.01 min and at 4% B for 7 min before the next injection. For TTX the gradient was: start at 0% B, 0%–15% B in 10 min, 15%–35% B in the next 12 min, back to 0% B at 32.01 min and 0% B for 5 min before the next injection.

The column eluate was mixed into a tee connector with the oxidant 100 mM H_3_PO_4_, 5 mM H_5_IO_6_, adjusted at pH 7.8 with 5 M NaOH, at a flow rate of 0.5 mL·min^−1^. The resulting mixture was heated while passing through a 1-mL knitted Teflon reactor coil (5 m × 0.50 mm i.d., Supelco, Madrid, Spain) immersed in a water bath at 75 °C. It was then acidified in another tee connector with 0.1 M HNO_3_ at a flow rate of 0.3 mL·min^−1^, to reach a detector outflow pH that should range between 5 and 7 [17]. The fluorescent eluted derivatives were monitored using a fluorescence detector at 330- and 395-nm excitation and emission wavelengths, respectively.

### 4.5. Method Validation

Method validation parameters were calculated for the separation of PST. Linearity was calculated by injecting mixtures of all standards at different concentrations in each selected matrix. The correlation coefficient (*r*) must not be lower than 0.99, as was recommended [53,54], and the results [22,23] were expressed as mg STX·diHCl eq·kg^−1^, where the toxicity equivalent factors (TEF) were taken from Oshima [20]. Results were obtained by mathematical treatment of the analysis data for each toxin at different concentrations, in the same manner as other authors have done previously [22,23].

The limit of detection (LOD) was determined for each matrix (mussel, clam, scallop and oyster) analyzing five replicate extracts of blank matrix repeated over 6 days (*n* = 30), calculating the signal-to-noise ratio at the corresponding retention time for each toxin, multiplying it by 3 and converting it into mg STX·diHCl eq·kg^−1^, as was mentioned before. The limit of quantitation (LOQ) was calculated multiplying LOD by a factor of 3 [50]. The limits of quantitation were also confirmed by spiking the blank matrices with the standards at the concentration of the calculated LOQ; the solutions were diluted until the signal-to-noise ratio was below 3, which corresponds to the limit of detection, and then multiplying it by 3 to obtain the limits of quantitation.

Repeatability was calculated for each matrix at 3 different levels of concentration (0.2 mg STX∙diHCl eq·kg^−1^, 0.8 mg STX∙diHCl eq·kg^−1^ and 1.6 mg STX∙diHCl eq·kg^−1^). Five replicates were analyzed and repeated over 3 days (*n* = 15), for each matrix at each concentration and for each standard. The same injections were used to calculate the retention times’ relative standard deviations (%RSD) for each toxin.

The percentage of recovery was determined analyzing five replicates repeated over 3 days for all toxins and matrices. This parameter was calculated at one level of concentration in each spiked matrix.

For TTX, when it was analyzed with the rest of PST in mussel matrix, the validation parameters were obtained as follows: linearity was calculated by injecting TTX standards diluted in water at different concentrations, ranging from 10.306 to 21.900 mg·kg^−1^; LOD was determined for mussel matrix by the analysis of five replicate extracts of blank matrix repeated over 6 days (*n* = 30), calculating the signal-to-noise ratio at the corresponding retention time for TTX and multiplying it by 3. LOQ was calculated by multiplying the LOD by a factor of 3 [50]. Recovery was calculated by the analysis of five replicate extracts of spiked mussel matrix, at one level of concentration.

### 4.6. Regeneration of Hypercarb Column and SPE Cartridges

To avoid TFA saturation in the Hypercarb^®^ column, water was run through it when the equipment was in standby mode. TFA can be adsorbed on the surface of porous graphitic carbon; therefore, after using this additive in the mobile phase, a regeneration of the column was undertaken to ensure the original Hypercarb^®^ selectivity of the column and that optimum performance was accomplished. Acid/base regeneration was suitable for ionized species being chromatographed in strongly-aqueous eluents. The regeneration recommended by the manufacturer [40] was as follows: the column was inverted at a flow rate of 1 mL·min^−1^ with 50 mL of 50% (*v*·*v*^−1^) tetrahydrofuran solution containing 0.1% (*v*·*v*^−1^) TFA, then flushed at 1 mL·min^−1^ with 50 mL of 50% (*v*·*v*^−1^) tetrahydrofuran solution containing 0.1% triethylamine or sodium hydroxide, followed by 50 mL of 50% (*v*·*v*^−1^) tetrahydrofuran solution containing 0.1% (*v*·*v*^−1^) TFA at a flow rate of 1 mL·min^−1^. Finally, 95% (*v*·*v*^−1^) methanol was eluted to re-equilibrate and re-invert the column. Before working back with the column, it was advisable to pump water through it overnight to remove any traces of solvents.

This regeneration was also useful for Hypercarb^®^ cartridges if they stop functioning, although this cartridge is stable throughout the entire pH range from 1–14, and it is not affected by aggressive solvents. This is the reason why Hypercarb^®^ cartridges could be used several times. After each usage, they were washed with water to remove citrate buffer. When this cleaning procedure was not enough, a thorough cleansing was made with more aggressive solvents.

## Figures and Tables

**Table 1:** Toxin linearity results: calibration curve equations and the corresponding correlation coefficients.

Compound	Range in mg·kg^−1^		Range in mg STX∙diHCl kg^−1^		Correlation (*r*)
	Lower	Upper	Lower	Upper	
dcNEO	0.021	1.33	0.009	0.575	0.9935
dcSTX	0.003	3.28	0.002	2.443	0.9849
dcGTX3	0.004	1.03	0.001	0.408	0.9987
STX	0.007	3.81	0.007	3.812	0.9950
NEO	0.052	1.66	0.046	1.816	0.9937
GTX5	0.160	2.62	0.010	0.165	0.9937
dcGTX2	0.002	4.37	0.0003	0.710	0.9982
GTX3	0.032	1.02	0.019	0.612	0.9986
GTX4	0.0007	1.28	0.0005	0.841	0.9911
GTX1	0.004	3.95	0.004	3.554	0.9994
C2	0.006	1.44	0.0005	0.109	0.9969
GTX2	0.043	2.77	0.014	0.934	0.9981
C1	0.157	5.01	0.0007	0.024	0.9980

**Table 2:** Limits of detection (LOD) and quantification (LOQ) for each toxin in each matrix (as mg STX∙diHCl kg^−1^).

Matrix		dcNEO	dcSTX	dcGTX3	STX	NEO	GTX5	dcGTX2	GTX3	GTX4	GTX1	C2	GTX2	C1
Mussel	LOD	0.0045	0.0004	0.0006	0.0044	0.0093	0.0027	0.0001	0.0012	0.0015	0.0037	0.0003	0.0001	0.0002
	LOQ	0.0070	0.0007	0.0019	0.0055	0.0163	0.0099	0.0003	0.0113	0.0059	0.0117	0.0007	0.0138	0.0005
Clam	LOD	0.0030	0.0003	0.0035	0.0041	0.0059	0.0007	0.0007	0.0114	0.0010	0.0008	0.0024	0.0043	0.0004
	LOQ	0.0040	0.0004	0.0035	0.0047	0.0078	0.0012	0.0014	0.0136	0.0026	0.0032	0.0027	0.0070	0.0005
Scallop	LOD	0.0344	0.0003	0.0002	0.0038	0.0054	0.0002	0.0004	0.0007	0.0007	0.0005	0.0001	0.0005	0.0001
	LOQ	0.0750	0.0004	0.0005	0.0039	0.0065	0.0007	0.0010	0.0018	0.0019	0.0011	0.0001	0.0012	0.0001
Oyster	LOD	0.0330	0.0003	0.0106	0.0039	0.0053	0.0069	0.0253	0.0062	0.0004	0.0016	0.0035	0.0366	0.0008
	LOQ	0.0750	0.0004	0.0111	0.0042	0.0065	0.0087	0.0258	0.0074	0.0009	0.0043	0.0037	0.0420	0.0008

**Table 3:** %RSD (Relative Standard Deviation) values for the repeatability of the method.

mg STX∙diHCl kg^−1^	Matrix	dcNEO	dcSTX	dcGTX3	STX	NEO	GTX5	dcGTX2	GTX3	GTX4	GTX1	C2	GTX2	C1
0.2	Mussel	7.7	5.1	2.7	4.7	8.7	6.4	5.9	3.8	2.4	2.9	4.0	4.6	5.6
	Clam	6.8	3.9	2.0	4.7	5.7	6.2	5.1	5.7	3.6	3.6	6.5	6.9	5.5
	Scallop	8.4	5.8	2.3	6.3	5.7	6.0	1.4	3.9	6.4	8.0	9.4	4.9	6.5
	Oyster	7.3	4.6	5.8	7.8	9.0	9.1	1.8	4.9	5.0	4.7	6.5	4.3	5.2
0.8	Mussel	3.9	4.6	3.6	5.3	4.8	2.8	5.7	4.4	5.5	2.7	6.1	5.5	4.1
	Clam	6.0	6.4	3.6	5.3	5.8	6.7	3.1	7.0	4.7	2.0	3.6	5.8	3.4
	Scallop	6.2	3.2	1.8	5.5	2.1	6.0	5.4	5.2	5.5	4.8	7.8	3.3	2.2
	Oyster	8.6	1.6	1.7	7.1	6.6	8.8	6.4	4.5	2.6	5.3	3.3	4.3	2.7
1.6	Mussel	2.3	4.3	6.4	5.6	4.5	1.7	2.6	4.7	5.4	2.1	4.6	2.7	3.7
	Clam	5.2	3.3	4.6	3.9	5.1	2.9	2.9	6.0	5.6	2.4	4.2	6.3	4.1
	Scallop	4.4	4.9	5.4	6.8	4.4	2.4	3.7	3.4	6.1	2.6	3.9	5.4	3.6
	Oyster	2.5	2.4	3.5	5.9	5.2	2.5	2.7	2.8	3.1	2.0	6.5	4.6	5.6

**Table 4:** %RSD values for the retention times (RT) of repeated injections of the PST (Paralytic Shellfish Toxins) standard solutions.

mg STX∙diHCl kg^−1^	Matrix	dcNEO	dcSTX	dcGTX3	STX	NEO	GTX5	dcGTX2	GTX3	GTX4	GTX1	C2	GTX2	C1
0.2	Mussel	0.8	0.6	0.3	0.8	0.8	0.7	0.6	0.5	0.4	0.3	0.4	0.4	0.2
	Clam	0.5	0.4	0.8	0.7	0.7	0.3	0.5	0.5	0.5	0.2	0.4	0.3	0.3
	Scallop	0.9	0.2	0.3	0.2	0.3	0.3	0.2	0.3	0.6	0.3	0.2	0.2	0.2
	Oyster	0.5	0.2	0.3	0.3	0.3	0.3	0.2	0.2	0.2	0.3	0.2	0.2	0.2
0.8	Mussel	0.6	0.5	0.4	0.1	0.2	0.3	0.3	0.3	0.3	0.6	0.2	0.2	0.5
	Clam	0.8	0.6	0.4	0.5	0.5	0.3	0.3	0.3	0.3	0.5	0.2	0.2	0.4
	Scallop	0.3	0.5	0.5	0.4	0.4	0.3	0.3	0.3	0.4	0.5	0.2	0.3	0.4
	Oyster	0.7	0.8	0.7	0.7	0.8	0.8	0.7	0.6	0.4	0.6	0.3	0.1	0.4
1.6	Mussel	0.7	0.6	0.4	0.5	0.4	0.7	0.8	0.4	0.7	0.5	0.5	0.5	0.8
	Clam	0.9	0.4	0.4	0.8	0.7	0.3	0.7	0.5	0.5	0.9	0.5	0.8	0.8
	Scallop	0.3	0.4	0.3	0.4	0.5	0.7	0.2	0.5	0.6	0.5	0.7	0.6	0.8
	Oyster	0.8	0.7	0.6	0.8	0.2	0.6	0.8	0.8	0.8	0.8	0.5	0.6	0.8

**Table 5:** Percentage of recovery (3 days, 5 replicates, *n* = 15) from all toxins in four matrices (mussels, clams, scallops and oysters).

Spiked Matrix		dcNEO	dcSTX	dcGTX3	STX	NEO	GTX5	dcGTX2	GTX3	GTX4	GTX1	C2	GTX2	C1
Matrix	Spiked level, mg·kg^−1^	0.08	0.03	0.02	0.04	0.09	0.16	0.08	0.05	0.02	0.06	0.03	0.13	0.10
Mussel	Recovery%	65.8	82.4	65.2	83.4	72	85.8	87.8	84.2	76.2	79.2	76	73.2	78
	%RSD	4.7	5.4	3.0	7.0	6.4	7.7	9.3	9.3	1.6	1.5	8.9	5.4	2.9
Clam	Recovery%	63.6	92.6	67.8	78.8	72.8	86.2	84	77.6	73	75.4	68.2	83	74.6
	%RSD	2.9	3.6	1.9	2.6	2.6	4.0	8.8	4.0	2.9	3.5	2.2	4.2	3.8
Scallop	Recovery%	72	88.2	69	80.2	72	84.6	118.6	78.6	74.4	73.8	75.2	82.6	73.8
	%RSD	2.1	1.3	1.2	1.9	2.5	2.9	1.7	1.5	3.6	4.1	2.9	4.0	2.5
Oyster	Recovery%	68.6	78.2	63.6	79.2	74.4	72.4	80	69.8	73.2	76	66.2	67.2	62.6
	%RSD	2.5	1.9	5.8	4.6	4.3	3.5	1.9	3.1	1.6	4.0	3.0	2.6	2.2
